# Camouflaging Intent, First Impressions, and Age of ASC Diagnosis in Autistic Men and Women

**DOI:** 10.1007/s10803-021-05221-3

**Published:** 2021-08-03

**Authors:** Hannah L. Belcher, Sharon Morein-Zamir, Will Mandy, Ruth M. Ford

**Affiliations:** 1 School of Psychology and Sport Science, Anglia Ruskin University, East Road, Cambridge, CB1 1PT UK; 2 University College London, 1-19 Torrington Place, Bloomsbury, London, WC1E 7HB UK; 3 Present Address: IOPPN, King’s College London, 16 Crespigny Park, Camberwell, London, SE5 8AB UK

**Keywords:** Autism, Camouflaging, Masking, First impressions, Female autism phenotype, Gender differences

## Abstract

Camouflaging of autistic traits may make autism harder to diagnose. The current study evaluated the relations between camouflaging intent, first impressions, and age of autism diagnosis. Participants comprised autistic and non-autistic adults (*n* = 80, 50% female) who completed the Camouflaging of Autistic Traits Questionnaire. They were later video-recorded having a conversation with a person unaware of their diagnostic status. Ten-second clips from half these videos were later shown to 127 non-autistic peers, who rated their first impressions of each participant. Results showed that autistic participants were rated more poorly on first impressions, males were rated less favourably than females, and male raters were particularly harsh in their evaluations of autistic males. Camouflaging intent did not predict first impressions but better first impressions were linked with a later age of diagnosis.

## Introduction

Not only is Autism Spectrum Condition (ASC) diagnosed much more commonly in males than females, with a male to female ratio of 3–4.5:1 (Baio, 2012; Bryson & Smith, 1998; Fombonne, 2003), but males generally receive their ASC diagnosis at an earlier age than females (Begeer et al., 2013). Indeed, most autistic females without intellectual impairment are diagnosed in adulthood (Baldwin & Costley, 2016), with one large-scale survey by the National Autism Society finding that only one fifth of autistic girls were diagnosed before the age of 11 years compared to over half of autistic boys (Bancroft, 2012). Regardless of whether there are genetic factors that reduce susceptibility to autism in females (e.g., Robinson et al., 2013; Skuse, 2000), it has been suggested that many autistic women experience delays in diagnosis because they show a phenotype of the condition with fewer overt autistic characteristics (the Female Phenotype Theory [FPT]; Kopp & Gillberg, 1992). For example, autistic females have been found in some studies to have a greater interest in social relationships than autistic males (Head et al., 2014), less obvious restrictive interests (Lai et al., 2011), and a tendency for distress to manifest as internalising disorders (e.g., depression, anxiety, and eating disorders) rather than externalising ones (Gotham et al., 2015; Oswald et al., 2016).

Consistent with the FPT, autistic females tend to make better first impressions than autistic males. This was first demonstrated by Cage and Burton (2019), who presented 10-s clips of 20 autistic adults and 20 non-autistic adults (10 female in each group) having a mock job interview to non-autistic observers who were unaware of ASC status. The non-autistic participants were rated more favourably overall (see also, Grossman, 2015; Sasson et al., 2017) but autistic females received significantly higher ratings than autistic males. In a similar study, Cola et al. (2020) arranged for 93 school-aged children, including autistic (62.5% male) and non-autistic (53% male) participants, to engage in a short conversation with a confederate; 21 undergraduate students fulfilled the role of conversational partner and were unaware of the true aims of the study. When the adults later evaluated children they had spoken to using the extended Conversation Rating Scale (CRS-E), they rated autistic girls significantly more positively than autistic boys and at a similar level to non-autistic girls, despite similar severity of autistic traits between boys and girls having been observed by clinicians on the Autism Diagnostic Observation Schedule (ADOS).

### Social Camouflaging

To some extent, the gender difference in overt signs of autism could reflect a greater tendency for females to employ *social camouflaging*, that is, learned behavioural strategies by which autistic people attempt to disguise and mask their condition in social contexts (Lai et al., 2015; Livingston & Happé, 2017). Masking features of one’s true self is not necessarily an autism specific phenomenon, for example, Goffman (1990) suggested that all humans adopt appropriate roles in social situations to control their appearance to others. However, this strategy is thought to be exaggerated in autistic people, thus requiring far more internal resources to maintain (Lai et al., 2015).

Early studies on camouflaging used qualitative methods to probe autistic individuals’ own understanding of what camouflaging involves (e.g., Hull et al., 2017; Tierney et al., 2016). In interviews, autistic females reported using deliberate mimicry during social interactions (e.g., Bargiela et al., 2016), as well as purposeful non-verbal gestures, maintaining appropriate levels of eye contact, avoiding dominating conversations, and practising conversations beforehand to maintain a social script (Hull et al., 2017). More recently, researchers have employed self-assessment measures to better conceptualise and assess autistic individuals’ *camouflaging behavioural intent*, that is, conscious intention to disguise autistic characteristics. For example, the self-report Camouflaging Autistic Traits Questionnaire (CAT-Q) was developed for use with adults (Hull et al., 2019). The 25 items in the CAT-Q were derived from earlier qualitative research (Hull et al., 2017). The scale measures three factors: (a) compensation, i.e., strategies to compensate for social and communication difficulties; (b) masking, i.e., strategies to appear less autistic to others; and (c) assimilation, i.e., strategies to blend or fit as seamlessly as possible into social situations. In an initial study, autistic adults (*n* = 306) were found to score significantly higher on the scale than non-autistic adults (*n* = 472) (Hull et al., 2019), while a follow-up study showed that autistic women (*n* = 182) scored significantly higher than autistic men (*n* = 108) on both the masking and compensatory factors of the scale, although not on the compensation factor (Hull et al., 2020).

In contrast, Cage and Troxell-Whitman (2019) found no significant difference between autistic males (*n* = 111) and autistic females (*n* = 135) on the CAT-Q. Instead, this study revealed a female advantage on an additional measure that examined the quality and context of camouflaging. Similarly, with a 4-item measure of camouflaging intent, Cassidy et al. (2018) did not find a significant difference in the percentage of autistic males (90.9%; *n* = 65) compared to autistic females (89.2%; *n* = 99) who reported trying to camouflage, but they did find evidence that females had a higher quality of camouflaging (*d* = 0.47). The scale asked participants if they had “ever tried to camouflage or mask [their] characteristics of ASC to cope with social situations? For example, have [they] ever tried to copy or mimic other people’s behaviour to try and fit in, or tried to mask or hide [their] symptoms of ASC from other people?” If participants answered affirmatively then they were asked to specify in which areas of their life they camouflaged, how frequently they did so, and the approximate amount of time per day they spent camouflaging. The findings suggested that while both autistic females and autistic males are motivated to camouflage, females may invest greater effort. Finally, a recent study found no difference between autistic males and females in self-reported compensatory behaviours (Livingston et al., 2020), consistent with the equivalent scores for autistic males and females on the compensation subscale of the CAT-Q as noted above in the study by Hull et al. (2020).

Autistic women may have stronger camouflaging intent than autistic men because they experience greater socialisation pressures from a young age to conform to gender stereotypes (Krahn & Fenton, 2012). Additionally, they might have an advantage over men in cognitive skills that support camouflaging, such as executive functions (EF) (Livingston et al., 2019). It has been suggested that EF could play an important role in the ability to inhibit inappropriate social responses, script social interactions in advance, and show flexibility in unexpected social situations (Sedgewick et al., 2016). Moreover, EF support an efficient Theory of Mind (ToM; the ability to infer other people’s thoughts, beliefs and feelings) (e.g., Ahmed et al., 2011) and ToM is likely to aid camouflaging by making it easier to judge how one is perceived by others (Hull et al., 2020b). Several studies have observed that autistic females exhibit better EF than autistic males (Bolte et al., 2011; Lai et al., 2012; Lenhardt et al., 2016), and Lai et al. (2017) found a positive correlation between self-reported camouflaging intent and cerebellum grey matter (associated with EF) in autistic women but not in autistic men. On the other hand, in the only study to date to have examined the contribution of ToM to camouflaging, Hull et al. (2021) found no reliable relation between ToM (as gauged by the Strange Stories task; Happé, 1994) and any component of camouflaging intent in autistic adolescents as gauged by the CAT-Q.

### The Present Study: Camouflaging Intent, First Impressions and Age of ASC Diagnosis

In summary, recent evidence suggests that autistic females make better first impressions than autistic males and are more likely to camouflage their autistic traits during social interactions. Such evidence is especially concerning given that the main tool used to diagnose autism, the ADOS, relies on clinical observations of social behaviours. Thus, camouflaging could, at least in part, explain the well documented delays that many autistic women experience in gaining an ASC diagnosis. To date, what is missing in the literature is any direct investigation of whether camouflaging intent is a significant predictor of first impressions by autistic individuals, regardless of gender, and whether camouflaging intent and/or first impressions predict age of ASC diagnosis. The primary aim of the present study was to conduct such an investigation. We predicted that for autistic men and women, (1) greater self-reported camouflaging intent would be associated with more positive first impressions, and (2) better first impressions would be associated with a later age of ASC diagnosis. Additionally, for the first time in the literature we assessed whether the first impressions made by autistic individuals, when rated by non-autistic peers who were uninformed that anyone they were evaluating was autistic, would differ between male and female observers. Females are widely reported to be more empathetic than males (review by Christov-Moore et al., 2014) and to be better at reading emotions (e.g., Hampson et al., 2006; McClure, 2000). These findings led us to expect that female raters would be more generous in their assessments of the participants overall, but especially in their evaluation of the autistic participants whom they may perceive as feeling more challenged by the task of making idle conversation with a stranger.

Our study was conducted in two phases. In Phase 1, autistic and non-autistic adults were invited to complete the CAT-Q, AQ, and measures of EF and ToM before being filmed in natural conversation with an unfamiliar person. In Phase 2, short segments of these video-recordings were viewed by a large sample of non-autistic peers who were asked to rate their first impressions of the video-recorded participants. Based on previous research, we had the following supplementary hypotheses. First, we predicted that autistic people would score higher on the CAT-Q than non-autistic people, while autistic females would score higher than autistic males (i.e., as reported by Hull et al., 2020). Second, it was anticipated that autistic adults would be rated less favourably in terms of first impressions than non-autistic adults (i.e., as reported by Sasson et al., 2017), and that autistic males would be rated less favourably than autistic females (i.e., as reported by Cage & Burton, 2019). Third, based on the arguments put forward by Hull et al. (2021), we expected that autistic participants who scored more highly on the tests of EF and ToM would also report greater camouflaging intent on the CAT-Q. Finally, because Hull et al. (2019) observed that CAT-Q scores were positively correlated with autistic traits as gauged by the Broader Autism Phenotype Questionnaire (BAPQ: Hurely et al., 2007), we assumed that the motivation to camouflage would be higher given a greater degree of autistic traits as gauged by the AQ.

## Method

### Participants

For Phase 1, participants were recruited via advertisements posted at local universities and on social media asking for young adults to take part in research looking at differences in social behaviours between autistic and non-autistic individuals. Most autistic participants were recruited from advertisements placed in private autism groups on Facebook and in community centres holding autism meetings or clinics. Participants were required to be UK citizens and speak English as a first language; this was to ensure that any cultural effects would not bias the second phase of the study, which would test the same group of participants. Participants were also required to be between the ages of 18–40 years to limit the effects of aging on autistic traits and EF. GPower 3.1.9.2 (small Cohen’s *d* effect size = 0.25, alpha = 0.05, df = 2, power = 0.8, and groups = 4) revealed 158 participants would be required for a two-way ANOVA. However, due to recruitment time constraints, a total of 80 participants was recruited; forty of these had an ASC diagnosis (20 males and 20 females) and the remainder were non-autistic controls (20 males and 20 females). One female and one male autistic participant identified as transgender and were grouped according to their currently identified gender. A post-hoc sensitivity analysis for a two-way ANOVA with 4 groups of 20 participants each, indicated our sample size would be able to detect moderate effects (Cohen’s *f*^2^ = 0.39, power = 0.80). Participants received £7 for their time (1 h) and all reasonable travel expenses were refunded.

ASC diagnoses were confirmed by requesting to see evidence, including education and health statements and diagnostic reports. Whilst all autistic participants reported having an ASC and details of how they were diagnosed, evidence was submitted by 29 participants. In most of the remaining 11 cases, the reports remained with their guardians as they were diagnosed as children (these cases were retained in our sample). Scores on the AQ screening measure did not differ significantly between autistic participants who had submitted evidence of their diagnosis (*M* = 35.09, *SD* = 7.65) and those who had not (*M* = 35.00, *SD* = 7.85), *t*(38) = 0.033, *p* = 0.974. Four of the latter group scored below the AQ criteria (> 32), however, three of these scored above the less conservative AQ criterion (> 28) suggested by Baron-Cohen et al. (2001) for those in clinical settings with an autism diagnosis. The video of the participant who scored below this criterion and did not provide a diagnostic report was not included in Phase 2 of the study. Autistic females received their diagnosis at a significantly later age (*M* = 22.25, *SD* = 10.00) than autistic males (*M* = 13.90, *SD* = 8.81), *t*(38) = 2.802, *p* = 0.008, *d* = 0.89.

None of the non-autistic participants reported an ASC diagnosis, although four reported having an autistic first-degree family member. We also checked whether any of them had other psychiatric diagnoses associated with externalised symptoms that might influence perceptions of their social behaviours, for example, bipolar disorder or psychosis. One participant reported having psychosis and their video-clip was not used in Phase 2. However, we retained the video-clips of participants who reported diagnoses of common mental health problems such as anxiety (*n* = 4), depression (*n* = 5), and OCD (*n* = 1) on the grounds that such problems tend to manifest internally. Reflecting the high comorbidity of other psychiatric disorders and autism (Russell et al., 2016), 50% of our autistic participants had another psychiatric diagnosis. We did not exclude their videos from Phase 2 to ensure a representative sample of the wider population of young autistic adults.

Table 1 shows, for each group, means and standard deviations for age, scores on the National Adult Reading Test (NART), and Autism Quotient (AQ) (see Measures section for full descriptions). The NART was administered as it is strongly correlated with IQ and previous research has shown IQ can affect camouflaging behaviours (Lehnhardt et al., 2016; Livingston & Happé, 2019). A series of two-way, between-group ANOVAs for diagnostic group (autistic versus non-autistic) and gender (male versus female) found no significant effects for either age or the NART, *p* values > 0.05. In line with the diagnostic group classification, autistic participants scored higher than non-autistic participants on the AQ, *F*(1, 76) = 86.675, *p* < 0.001, η_p_^2^ = 0.533. There was no significant effect of gender on the AQ and no significant group x gender interaction, *p* values > 0.05.

**Table 1:** Group means and standard deviations for age and scores on the NART and AQ

Measure	ASC		Non-Autistic	
	Females	Males	Females	Males
Age	25.45 (7.9)	25.85 (6.06)	27.75 (5.82)	27.80 (5.94)
NART	112.11 (3.53)	110.89 (6.17)	110.53 (3.44)	111.00 (4.08)
AQ	36.55 (7.55)	33.50 (7.73)	18.25 (8.99)	18.90 (7.22)

For Phase 2, participant-raters were recruited from the university, using both online and physical posters, for a study looking at social judgements of others based on first impressions. Course credits were offered, as well as a place in a prize draw with a chance to win a £50 Amazon voucher. Power analysis using GPower 3.1.9.2 with a small effect of 0.3 (Cohen’s *d*), an alpha level of 0.05, and a power level of 0.8, as for Sasson et al. (2017), determined that a sample size of 58 would be sufficient for a repeated measures ANOVA with four measures. In total, 53 males and 74 females were recruited; one male was transgender and categorised according to their identified gender. Participants were aged 18 to 40 years (males: *M* = 27.17, *SD* = 6.05, females: *M* = 24.08, *SD* = 5.51). They were required to have no ASC, or any uncorrected visual or hearing impairments, and to speak English as their first language. These criteria ensured the participant-raters were similar to the participants being observed (hereafter referred to as participant-stimuli) in terms of age and cultural background.

### Measures

#### National Adult Reading Test (NART)

The NART (Nelson & Willison, 1991) comprises a list of 50 words which become progressively harder to pronounce as the list goes on. Participants are instructed to read each of the words on the list aloud, and a point is assigned if the word is pronounced correctly. NART error scores are used to predict WAIS full scale IQ, verbal IQ, and predicted IQ (Bright et al., 2018).

#### Autism Quotient (AQ)

The full 50-item AQ was used to measure autistic traits. Analysis has revealed five factors commonly observed to be atypical in autistic participants (communication, social, imagination, local details, and attention switching), which collectively have an acceptable Chronach’s alpha coefficient (0.67). The AQ is reported to have good test–retest reliability (*r* = 0.7) and a cut off score of ≥ 32 has been found to be accurate in identifying likely cases of ASC in the general population (Baron-Cohen et al., 2001).

#### Camouflaging of Autistic Traits Questions (CAT-Q)

The Camouflaging Autistics Traits Questionnaire (CAT-Q) is a 25-item self-report questionnaire developed from the theoretical model set out by Hull et al. 2017), who provided a qualitative analysis of camouflaging by autistic participants. The items in the questionnaire were intended to reflect two aspects of camouflaging: first, compensation of social and communication difficulties, and second, masking one’s presentation to appear non-autistic (Hull et al., 2019). Participants answer each question on a seven-point Likert scale from ‘Strongly Disagree’ to ‘Strongly Agree’, with higher scores indicating stronger camouflaging intent. The scale was validated by the authors on 354 autistic participants and 478 non-autistic participants (300 males and 434 females) with a mean age of 36. Factor analysis revealed that the scale measured three factors: compensation and masking (as described above), and assimilation, which involved strategies reflecting a need to fit in with others socially. High internal consistency was found by the authors for the scale as a whole (α = 0.94), as well as each of the three subscales (Compensation = 0.91, Masking = 0.85, and Assimilation = 0.92). Test–retest reliability, as calculated from 30 autistic participants who completed the questionnaire again three months later, was high (*r* = 0.77). Furthermore, convergent validity was achieved because outcomes for the CAT-Q were significantly, positively correlated with autistic traits and social anxiety in both autistic and non-autistic samples, and with depression and generalised anxiety in autistic participants (non-autistic participants were not tested with depression and anxiety measures) (Hull et al., 2019).

#### Executive Functioning (EF)

A battery of EF tasks was administered using PEBL software (Mueller & Piper, 2014). The tasks comprised (1) Berg’s ‘Wisconsin’ Card Sorting Test (BCST; Berg, 1948) to measure set-shifting, (2) the Tower of London task (TOL; Shallice, 1982) to measure planning, and (3) the Numerical Stroop task (Stroop, 1935; Windes, 1968) to measure inhibition.

#### The Short Story Task (SST)

The SST is a 14-question task measuring both first-order ToM (understanding another person’s thoughts) and second-order ToM (understanding what another person is thinking about another person’s thoughts). Inter-rater reliability has been found to be relatively high for both mental state reasoning (0.98) and comprehension (0.90) (Dodell-Feder et al., 2013). After reading a short extract from the story ‘The End of Something’ by Ernest Hemingway, participants answer one question measuring spontaneous mental state reasoning (scored 0–1), five questions measuring comprehension (each scored 0–2), and eight questions measuring explicit mental state reasoning (each scored 0–2).

#### First Impressions Scale

This questionnaire, which was compiled from previously developed instruments for the purposes of the present study, had 10 items on which the participant-raters judged the social behaviours of the participant-stimuli, and their own behavioural intentions towards the participant-stimuli, on a 4-point scale from ‘strongly agree’ to ‘strongly disagree’ (four items reverse-scored) (see Appendix 1). Higher first impression scores indicate more favourable assessments. There were six items found previously to reliably measure first impressions of the participant-stimuli’s social behaviours (i.e., *traits*), for example, social awkwardness (Grossman, 2015; Willis & Todorov, 2006) and four items found previously to reliably measure the intentions of the participant-raters regarding potential future dealings with the participant-stimuli (i.e., *behavioural intentions*), for example, willingness to start a conversation with the person (Campbell et al., 2004; Matthews et al., 2015; Nevill & White, 2011). Sasson and Morrison (2019) showed that these 10 items could be averaged into an overall score for *first impressions* that had strong internal consistency (α = 0.82).

### Procedure

In Phase 1, the 80 participant-stimuli first completed an online survey, which included the CAT-Q and AQ, alongside questions about their age, gender, and formal ASC diagnosis status. Participants then attended a one-hour laboratory session where they re-consented and were filmed having an everyday conversation with one of two female research assistants, aged in their 20 s, who were not informed that any of the participants were autistic. Following procedures used by Sasson et al. (2017), the participant-stimuli were recorded engaging in a natural conversation. The research assistant sat directly opposite the participant and initially asked open-ended questions about mundane topics to establish rapport, assuring them that their replies weren’t being evaluated. To ensure consistency of content across participant-stimuli, the research assistants asked at a natural and convenient point in the conversation if the participant could describe a film or book they had recently watched or read, or that was their favourite. Each research assistant wore a GoPro camera (Hero 4; recording in 1080p wide at 60fps) on their head to record the conversation from a first-person perspective. Following the video recording, participant-stimuli completed the computer battery of EF tasks, in random order, in addition to the NART and SST.

For Phase 2, 10–15 s clips were extracted from the participant-stimuli videos (one autistic male did not consent to filming). These were taken whilst the participant discussed a book or film, which occurred midway or towards the end of the conversation. Two independent raters, who were blind to group status, reviewed the clips to ensure consistency in sound and picture quality, identifying 18 clips deemed of insufficient quality. A further 6 clips were discarded due to participants’ visible disabilities or strong regional accents. The final selection comprised clips for 15 non-autistic females, 19 non-autistic males, 14 autistic females, and 14 autistic males. Of these, ten clips were randomly selected from each of the four participant-stimuli groups.

Participant-raters completed the study either online or in a university laboratory. Participant-raters were informed that the study would involve watching and listening to 40 video-recordings of individuals engaged in a conversation and then rating the individuals’ social impressions. They were not informed that some of the videos were of autistic people. After providing informed consent, participants keyed in a password provided in a short test video to verify audio-visual quality. Five videos from each of the four participant-stimuli groups were played randomly, followed by the opportunity for a five-minute break, and then the final 20 videos. The first impressions scale was presented after each video.

The study was approved by the university research ethics committee. Informed consent was gained twice from participant-stimuli (before the online portion and again before the laboratory session). Participant-raters also provided informed consent and were asked to ensure the confidentiality of all participant-stimuli they observed. No personal or diagnostic details were provided of participant-stimuli, and videos could not be downloaded or saved. All participants were fully debriefed and given the right to withdraw their data within the next 6 months.

## Results

Results are presented in two sections. In a preliminary analysis, we tested our supplementary hypotheses relating to Phase 1 of the study, namely, the effects of diagnostic group and gender on camouflaging intent, EF and ToM, and correlations between scores for the CAT-Q, AQ, EF and ToM (all of which have been examined in previous research). In our main analysis, we addressed our primary hypotheses relating to, first, the associations between camouflaging intent, first impressions, and age of ASC diagnosis, and second, the effects of rater gender on first impressions.

### Preliminary Analysis

#### Effects of Diagnostic Group and Gender on Camouflaging Intent, EF, and ToM

Table 2 presents descriptive statistics (means and standard deviations/frequency data) for all measures as a function of gender and group. Overall scores for EF and ToM were derived by converting results for the relevant component measures to *z* scores and averaging them, with higher values representing better performance. A series of two-way, between-group ANOVAs for diagnostic group (autistic versus non-autistic) and gender (male versus female) were conducted on the CAT-Q, EF and ToM scores. These analyses included all 80 participant-stimuli described previously, apart from ToM for which four participants had missing data (one in each of the four groups). Data were screened using visual inspection of distribution histograms together with Kolmogorov–Smirnov tests to detect departures from normality. BCST scores from the EF battery were subsequently log transformed to reduce skew.

**Table 2:** Means and standard deviations on all measures as a function of group and gender

Measure	ASC		Non-Autistic	
	Females	Males	Females	Males
CAT-Q	123.20 (28.76)	114.47 (27.06)	89.95 (25.69)	88.90 (29.36)
Compensating	42.60 (12.68)	39.53 (11.40)	26.10 (10.94)	25.80 (12.46)
Masking	38.50 (11.17)	34.58 (11.93)	35.60 (10.42)	35.50 (7.26)
Assimilation	42.05 (12.25)	40.37 (8.45)	28.20 (8.76)	27.60 (12.29)
ToM (*z* score)	− .03 (.74)	− .09 (.83)	− .02 (.74)	.14 (.74)
Inferences	10.53%	21.05%	10.53%	26.32%
Comprehension	68.42 (17.72)	65.79 (19.53)	66.32 (16.06)	72.63 (17.90)
Mental state	49.67 (14.80)	41.78 (17.44)	51.97 (18.05)	49.67 (17.98)
EF (*z* score)	− 0.01 (0.56)	− 0.21 (1.04)	0.23 (0.41)	− 0.01 (0.62)
Stroop interfere	3% (5%)	11% (19%)	5% (4%)	8% (7%)
BCST % correct	81.25 (7.35)	78.74 (12.85)	76.57 (11.28)	76.70 (13.01)
ToL	22.80 (8.67)	23.70 (8.25)	26.70 (6.07)	25.20 (7.93)

#### CAT-Q

A two-way ANOVA on the overall scores revealed a significant main effect for diagnostic group, reflecting greater camouflaging intent in the autistic participants, *F*(1, 76) = 23.017, *p* < 0.001, η_p_^2^ = 0.23. The main effect for gender was not significant, *F*(1, 76) = 0.580, *p* = 0.449, nor was the interaction between gender and group, *F*(1,76) = 0.350, *p* = 0.556.

#### EF

A two-way ANOVA on the overall EF *z* scores uncovered no significant effect of diagnostic group, *F*(1, 76) = 2.112, *p* = 0.150, η_p_^2^ = 0.03, no significant effect of gender, *F*(1, 76) = 1.999, *p* = 0.161, η_p_^2^ = 0.03, and no significant interaction, *F*(1, 76) = 0.016, *p* = 0.898, η_p_^2^ = 0.00.

#### ToM

A two-way ANOVA on the overall ToM *z* scores revealed no significant main effect for diagnostic group, *F*(1, 72) = 0.479, *p* = 0.491, η_p_^2^ = 0.01, no significant main effect for gender, *F*(1, 72) = 0.089, *p* = 0.767, η_p_^2^ = 0.00, and no significant interaction, *F*(1,72) = 0.441, *p* = 0.509, η_p_^2^ = 0.01.

#### Correlates of Camouflaging Intent

There were no significant correlations between any of the CAT-Q scales and either EF or TOM, regardless of group; autistic group range = -0.05 to 0.14, non-autistic group range = -0.17 to 0.11, all *p* values > 0.05. Likewise, there were no significant correlations between the AQ and either EF or TOM, regardless of group; autistic group range = -0.14 to 0.20, non-autistic group range = -0.19 to -0.03, all *p* values > 0.05. However, the autistic group showed a significant, positive correlation between CAT-Q Assimilation and AQ scores, *r*(40) = 0.54, *p* < 0.001. Likewise, the non-autistic group showed significant, positive correlations between AQ scores and both CAT-Q Compensation, *r*(40) = 0.37, *p* = 0.020, and CAT-Q Assimilation, *r*(40) = 0.45, *p* = 0.003, as well as overall CAT-Q, *r*(40) = 0.40, *p* = 0.011. Following Bonferroni correction, the correlation between the AQ and CAT-Q Assimilation remained significant in both groups (*p* < 0.006).

### Main Analysis

#### Camouflaging Intent and Age of ASC Diagnosis

To see whether autistic participants who reported greater camouflaging intent received their ASC diagnosis at an older age, correlations were calculated between the CAT-Q subscales and age of ASC diagnosis, separately for males and females. Spearman’s correlations were used due to non-normal distributions. Correlations for females were small to negligible; range = -0.22 to 0.03, *p* values > 0.05. Correlations for males were small to moderate and none survived Bonferroni correction (*p* values > 0.008); Compensation *r*_*s*_(20) = 0.34, *p* = 0.141, Masking *r*_*s*_(20) = 0.21, *p* = 0.366, and Assimilation *r*_*s*_(20) = 0.44, *p* = 0.05.

#### Camouflaging Intent and First Impressions

Spearman’s correlations were calculated to examine the associations between scores for CAT-Q scales and first impressions. When the whole video-clipped sample was considered (*n* = 40), all correlations were negligible or small, range = -0.07 to 0.29, *p* values > 0.05. When each diagnostic group was considered separately, none of the correlations in the autistic group survived Bonferroni correction; Compensation *r*_*s*_(20) = 0.41, *p* = 0.075, Masking *r*_*s*_(20) = 0.49, *p* = 0.028, Assimilation *r*_*s*_(20) = 0.03, *p* = 0.892. In the non-autistic group, correlations were similarly non-significant and were negligible or small, range = -0.02 to 0.28, *p* values > 0.05.

#### First Impressions and Age of ASC Diagnosis

Spearman’s correlations were calculated to examine the associations between first impressions and age of ASC diagnosis. There was a significant, positive correlation between first impressions and age of ASC diagnosis, *r*_*s*_(20) = 0.60, *p* = 0.005. When considered separately for each gender, the association was non-significant for autistic females, *r*_*s*_(10) = 0.22, *p* = 0.550, but remained significant for autistic males, *r*_*s*_(10) = 0.72, *p* = 0.019.

#### Effects of Rater Gender on First Impressions

For the analyses of first impressions, the participants were the raters and the stimuli were the four groups of ratees (i.e., autistic females, autistic males, non-autistic females, and non-autistic males). The dependent variable was the overall first impressions scores. Table 3 shows descriptive statistics for overall scores (i.e., summing results for behavioural intent and traits), broken down by diagnostic group and the gender of the participant stimuli. Results were evaluated using a (2 × 2) × 2 mixed ANOVA for participant-stimuli diagnostic group (autistic versus non-autistic), participant-stimuli gender (male versus female) and participant-rater gender (male versus female). Distributions of first impression scores were normal for each group, and Levene’s test was non-significant.

**Table 3:** Means and standard deviations of first impressions as a function of group and gender

	Autistic females	Autistic males	Non-autistic females	Non-autistic males
First-impressions	28.06 (2.70)	26.78 (2.91)	29.45 (2.85)	28.67 (3.06)
Behavioural-intent	11.28 (1.37)	10.83 (1.53)	11.98 (1.43)	11.57 (1.70)
Live near^a^	3.01 (0.42)	3.11 (0.53)	3.28 (0.48)	3.21 (0.50)
Hang out	2.48 (0.45)	2.29 (0.45)	2.66 (0.44)	2.54 (0.46)
Sitting next to^a^	3.14 (0.54)	2.94 (0.48)	3.21 (0.54)	3.11 (0.55)
Start conversation	2.65 (0.45)	2.49 (0.48)	2.82 (0.44)	2.71 (0.46)
Traits	16.74 (1.53)	15.92 (1.59)	17.45 (1.64)	17.05 (1.70)
Socially awkward^a^	2.34 (0.43)	2.20 (0.44)	2.85 (0.39)	3.05 (0.40)
Attractive	2.58 (0.44)	2.07 (0.44)	2.59 (0.42)	2.53 (0.43)
Trustworthy	2.91 (0.31)	2.86 (0.33)	2.96 (0.32)	2.82 (0.35)
Aggressive^a^	3.25 (0.42)	3.25 (0.42)	3.11 (0.46)	2.91 (0.48)
Likeable	2.88 (0.32)	2.78 (0.35)	3.06 (0.32)	2.96 (0.33)
Smart	2.78 (0.48)	2.76 (0.55)	2.88 (0.48)	2.77 (0.49)

^a^Reverse scored item as negatively worded (higher score = more favourable)

Significant main effects were found for participant-stimuli group, *F*(1,123) = 147.498, *p* < 0.001, η_p_^2^ = 0.55, participant-stimuli gender, *F*(1,123) = 55.110, *p* = 0.001, η_p_^2^ = 0.31, and participant-rater gender, *F*(1,123) = 8.369, *p* = 0.005, η_p_^2^ = 0.08. As can be seen from Table 3, autistic participant-stimuli were rated less favourably than non-autistic participant-stimuli, males were rated less favourably than females, and male participant-raters evaluated all groups more negatively than did female participant-raters.

A significant 2-way interaction was found between participant-stimuli group and participant-stimuli gender, *F*(1,123) = 11.086, *p* = 0.001, η_p_^2^ = 0.08, reflecting a greater female advantage in first impression ratings in the autistic group. However, non-significant interactions were found between participant-stimuli group and participant-rater gender, *F*(1,123) = 0.345, *p* = 0.558, η_p_^2^ = 0.03, and between participant-stimuli gender and participant-rater gender, *F*(1,123) = 0.326, *p* = 0.5691, η_p_^2^ = 0.03.

**Fig. 1:**
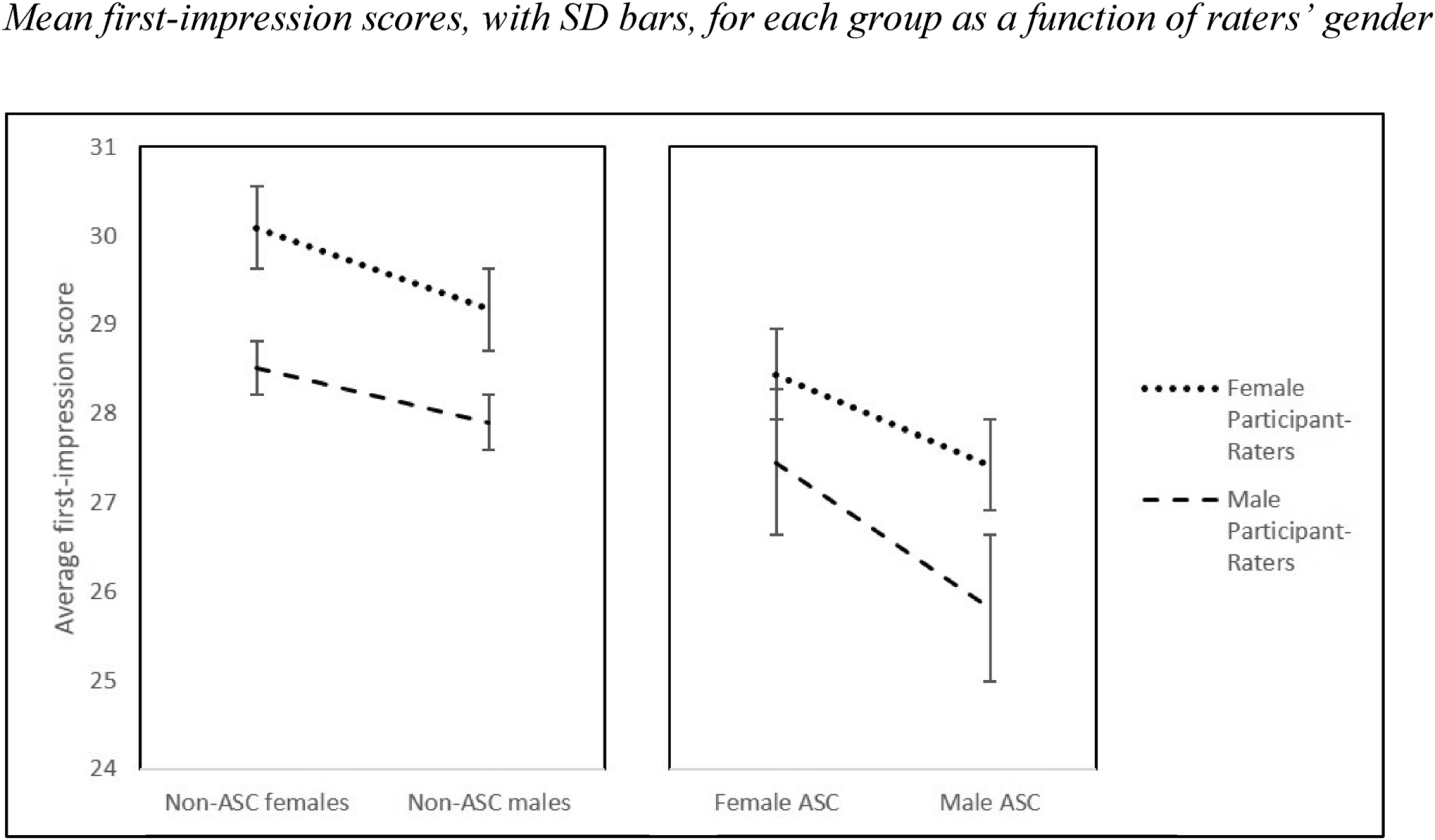
Mean first-impression scores, with SD bars, for each group as a function of raters’ gender

Importantly, the 3-way interaction between participant-stimuli group, participant-stimuli gender and participant-rater gender was also significant, *F*(1,123) = 5.444, *p* = 0.021, η_p_^2^ = 0.42. This interaction was followed with two (2 × 2) simple repeated-measure ANOVAs that explored the interaction between participant-stimuli gender and participant-stimuli group separately for male and female raters (see Fig. 1).

For female raters, there was no significant interaction between gender and group, *F*(1,72) = 0.679, *p* = 0.413, η_p_^2^ = 0.009. Overall, females rated autistic participants less positively than non-autistic participants, *F*(1,72) = 101.880, *p* < 0.001, η_p_^2^ = 0.586, and males less positively than females, *F*(1,72) = 53.920, *p* < 0.001, η_p_^2^ = 0.428. Male raters likewise showed main effects for both group, *F*(1,51) = 53.855, *p* < 0.001, η_p_^2^ = 0.514, and gender, *F*(1,51) = 16.354, *p* < 0.001, η_p_^2^ = 0.243. However, they also showed a significant interaction between group and gender, *F*(1,51) = 11.716, *p* = 0.001, η_p_^2^ = 0.187, reflecting particular harsh evaluations of autistic males. Bonferroni-corrected paired *t* tests revealed that autistic males (*M* = 25.81, *SD* = 2.79) were rated less positively than autistic females (*M* = 27.45, *SD* = 2.33), non-autistic males (*M* = 27.90, *SD* = 2.96), and non-autistic females (*M* = 28.51, *SD* = 2.40), *p* values < 0.001, while first-impression scores failed to differ reliably between autistic females and non-autistic males or between non-autistic males and non-autistic females, *p* values > 0.05.

## Discussion

It has been suggested that a strategy of camouflaging, that is a deliberate effort to disguise autistic behaviours, could be a contributing factor to the typically older age of ASC diagnosis in autistic women compared to autistic men (Lai et al., 2011). This being the case, it follows that camouflaging intent, first impressions, and age of ASC diagnosis should be positively correlated in the autistic population. The primary aim of this study was to evaluate this proposition by assessing camouflaging intent in autistic and non-autistic men and women using the self-report CAT-Q and examining, first, whether higher CAT-Q scores were associated with better ratings for first impressions by non-autistic peers, and second, whether higher CAT-Q scores and/or more favourable first impressions were predictive of a later age of ASC diagnosis. We also compared first impressions of autistic versus non-autistic participants between male and female raters, predicting that females would give more favourable evaluations than males for both diagnostic groups, but would be less severe on the autistic participants especially.

Against expectations, we did not find strong evidence that camouflaging intent predicted either first impressions or age of ASC diagnosis in the autistic group. This was despite the finding that, consistent with Hull et al. (2019), the autistic group scored higher on camouflaging intent than the non-autistic group, reinforcing the notion that camouflaging intent is a real phenomenon of relevance to understanding the experiences of autistic people. As suggested by Lai et al. (2017), many autistic individuals may attempt to camouflage during social interactions with the goal of blending in, avoiding possible discrimination and stigmatisation, and improving their employment and career prospects. The lack of beneficial impact of camouflaging intent on first impressions could mean that autistic participants lacked insight into their camouflaging abilities, thus self-reporting these as better than they really were. Alternatively, it could reflect the fact that CAT-Q scores, especially for assimilation, were significantly greater among participants with stronger autistic traits. It seems that despite their best efforts, such individuals may not have been able to conceal the more salient traits of their autism. Similar observations regarding a positive association between the CAT-Q and the severity of autistic traits were reported by Hull et al. (2019) and, like our study, this trend was evident for both autistic and non-autistic samples. Because research on the broader autism phenotype indicates that it is characterised by pragmatic language difficulties, impaired social functioning, and behavioural/cognitive inflexibility (Wainer et al., 2011), our results suggest that even non-autistic individuals with elevated levels of autistic traits are aware of their social difficulties and seek to compensate for them.

It is unlikely that autistic individuals with stronger autistic traits failed to turn their high camouflaging intent into effective camouflaging behaviours due to difficulties with EF, ToM, or both. This is because the groups did not differ significantly in EF and ToM, and there were no significant correlations between EF, ToM, and AQ scores in either case. Instead, it could be that high camouflaging intent among autistic participants with a higher degree of autistic traits was countered by impairments of *spontaneous* mimicry. Research with non-autistic adults has revealed that social interactions are facilitated by unconscious behaviours such as automatic mimicking of others’ postures, facial expressions, and mannerisms (i.e., the *chameleon effect*; Chartrand & Bargh, 1999), and there is evidence that autism may be characterised by impairments of spontaneous mimicry, particularly in relation to facial expressions (Oberman et al., 2009; Stel et al., 2008). Interestingly, in contrast to suggestions about deliberate, conscious masking of autistic traits, Lawson (2020) recently characterised camouflaging as ‘adaptive morphing’, that is, a defensive reaction designed to protect an autistic person from social trauma that may occur quite automatically. Analysis of responses from a large sample of autistic individuals, both male and female, indicated that many were mindful of wanting to fit in socially but not necessarily of actively trying to deceive other people. Lawson (2020) therefore speculated that a life-long motivation to avoid social exclusion might result in some spontaneous adaptation and remediation of the biological systems underpinning social interaction. Similarly, Bargiela et al. (2016) suggested that camouflaging develops in childhood as a coping strategy and can include both aware and unaware elements.

But while strength of camouflaging intent in autistic individuals did not predict whether they made a good first impression, as hypothesised, better first impressions *were* linked with a significantly later age of ASC diagnosis. This was true for the autistic group as a whole and was strongly evident even within the male subgroup. These findings suggest that aspects of social functioning weigh heavily with clinicians who are considering the possibility of an ASC diagnosis, potentially downplaying the significance of autistic traits in non-social domains or, alternatively, prompting attribution of such traits to other conditions such as Obsessive Compulsive Disorder and Borderline Personality Disorder (Lai & Baron-Cohen, 2015).

One of the clear implications for diagnostic practices used to identify ASC is that clinicians, teachers, and relevant health- and social care professionals should be educated about the possibility of seemingly unimpaired social behaviours of autistic individuals without intellectual impairment, particularly females, to ensure that autistic traits in non-social domains are not overlooked. That is, signs that an individual engages as expected in social interaction and makes a fair first impression should not negate an autism diagnosis if other traits of ASC are present. Indeed, the importance of this is underscored by evidence that timely diagnosis and support is vital for an autistic person’s mental health and wellbeing. Fernell et al. (2013) pointed out that earlier diagnosis facilitates the creation of a more autism-friendly environment around an autistic person, while qualitative studies have indicated that many autistic people feel relieved to receive their ASC diagnosis because it helps them to make sense of their experiences (Stagg & Belcher, 2019). Furthermore, prompt diagnosis enables the introduction of early interventions for autistic individuals that can greatly improve their quality of life (Howlin, 1997).

Finally, when comparing the first impressions reported by male versus female raters, our predictions were partly supported. Based on evidence of a female empathy and emotion-reading advantage (see Christov-Moore et al., 2014; McClure, 2000), we had anticipated that female raters would be more generous than male raters in their evaluations across all four groups, but especially for the autistic men and women. Although we did, indeed, observe that female raters gave significantly more positive appraisals than male raters overall, they were not unduly lenient for autistic participants. Thus, higher evaluations for the non-autistic group over the autistic group were not moderated by participant-rater gender. Instead, as reflected in a three-way interaction, female magnanimity was exaggerated only for autistic *males.* Specifically, the first impressions made by autistic males were judged particularly harshly by male raters compared to female raters. Clearly, further research is needed to determine whether this gender difference in first impressions of autistic males is underpinned by raters’ affective or cognitive empathy, ability to perceive emotions in others, or some other variable unrelated to emotional rapport. Regardless of reasons, though, it will also be important to establish whether the same pattern of findings holds up for clinicians who are fully aware that the people they are evaluating could be autistic. Autism is commonly diagnosed by a psychiatrist and until recently the majority of psychiatrists in the UK were male (NHS, 2018). This may have contributed to the historical bias of diagnosing males with autism at an earlier age than females, beyond any influence of either the female phenotype or tendency to perceive autism as a predominantly male disorder.

## Summary and Conclusions

In summary, we explored for the first time the associations between camouflaging intent, first impressions, and age of diagnosis in autistic men and women. Results showed that while self-reported camouflaging intent as gauged by the CAT-Q was not strongly correlated with first impressions, individuals who made more favourable first impressions tended to report a later age of ASC diagnosis. Our study also produced the novel finding that first impressions of autistic males were particularly unfavourable when they were rated by males rather than females.

Because we recruited autistic participants from the general population rather than assessment clinics, we were able to engage more individuals with non-traditional diagnosis records than was typical in previous research. Moreover, our advertisement purposefully did not mention that the study was exploring camouflaging behaviours, to avoid receiving a biased sample of high camouflaging participants. Despite these efforts to gain a representative sample of autistic adults, however, our participants were diagnosed much later on average (18.08 years) than the usual age reported in the literature (3–10 years; Daniels & Mandell, 2014). This may explain our failure to replicate some findings from previous research. Specifically, in our preliminary analysis we failed to find a female advantage in either CAT-Q scores or EF, and we obtained no significant correlation between camouflaging intent and EF.

Follow-up studies should seek to improve understanding of camouflaging and its role in first impressions in several ways. First, camouflaging intent should be assessed with multiple converging measures, including measures of how often participants are aware of engaging in camouflaging and the situations or circumstances in which they mainly use it. Second, participants could be asked to report their actual camouflaging strategies immediately following situations for which first impressions are measured, thus supplying direct evidence for any objective effectiveness and utility of specific camouflaging attempts. Third, given our observation that camouflaging intent was not predictive of first impressions, it may be fruitful to explore the extent to which first impressions depend more on the kinds of unconscious imitation underpinning the chameleon effect. If autistic women have an advantage over autistic men in this respect, then this could constitute an additional dimension of the female phenotype that helps to conceal their autism and make diagnosis harder.

It has been proposed that one important benefit of gaining an ASC diagnosis is improved self-understanding and ability to contextualize a lifetime of experiences that contribute to one’s identity (Leedham et al., 2019). Further to this, it has been argued that for many people an autism diagnosis has the positive effect of helping them to find a place in a community of autistic individuals who understand and support one another Kapp (2020). Coupled with our finding that autistic individuals’ deliberate attempts to disguise their condition may not be effective in altering the impression they make on others during social interactions, this suggests that they should instead be encouraged to embrace an autistic identity and form connections with like-minded people in an authentic way. Giving emphasis to this conclusion, it has been reported that intentional camouflaging behaviours are linked with depression, anxiety, and even suicidal behaviours, highlighting the psychological burden of pretending to be something one is not (Cassidy et al., 2018).

Additionally, there is a need to raise awareness of autism, and its wide range of manifestations in both men and women, within the general population. Milton (2012) highlighted a double empathy problem existing between autistic and non-autistic people, whereby not only are autistic people impaired at recognising non-autistic people’s behavioural intentions and feelings, but the reverse is also true. Some studies of first impressions made by autistic people have shown that when non-autistic observers are informed that the video-clips they are judging are of individuals with an ASC diagnosis, they rate them more favourably than when they are unaware of the diagnosis (Matthews et al., 2015; Sasson & Morrison, 2019). Thus, interventions to increase knowledge about the diversity of social behaviours exhibited by autistic people could lead to greater tolerance of individuals who present atypically, and ultimately reduce the pressure that autistic people so often perceive to blend in and hide their condition.

## Appendix: First Impression Scale

Please indicate how much you agree or disagree that you would:

**Table:**

	Strongly Agree	Agree	Disagree	Strongly Disagree
Mind if you had to live near this person				
Hang out with this person in your free time				
Be uncomfortable sitting next to this person				
Start a conversation with this person				

Please indicate how much you agree or disagree that this person is:

**Table:**

	Strongly Agree	Agree	Disagree	Strongly Disagree
Socially awkward				
Attractive				
Trustworthy/honest				
Aggressive/dominant				
Likeable				
As smart as you				
